# Efficient cellular fractionation improves RNA sequencing analysis of mature and nascent transcripts from human tissues

**DOI:** 10.1186/1472-6750-13-99

**Published:** 2013-11-13

**Authors:** Ammar Zaghlool, Adam Ameur, Linnea Nyberg, Jonatan Halvardson, Manfred Grabherr, Lucia Cavelier, Lars Feuk

**Affiliations:** 1 Department of Immunology, Genetics and Pathology, Rudbeck Laboratory and Science for Life Laboratory, Uppsala University, Uppsala, Sweden; 2 Department of Medical Biochemistry and Microbiology, Science for Life Laboratory, Uppsala University, Uppsala, Sweden

**Keywords:** RNA sequencing, Transcriptomics, RNA splicing, RNA purification, PolyA+ selection, Cytoplasmic RNA, Nuclear RNA, Nascent transcripts, De novo assembly, Transcription profiling

## Abstract

**Background:**

The starting material for RNA sequencing (RNA-seq) studies is usually total RNA or polyA+ RNA. Both forms of RNA represent heterogeneous pools of RNA molecules at different levels of maturation and processing. Such heterogeneity, in addition to the biases associated with polyA+ purification steps, may influence the analysis, sensitivity and the interpretation of RNA-seq data. We hypothesize that subcellular fractions of RNA may provide a more accurate picture of gene expression.

**Results:**

We present results for sequencing of cytoplasmic and nuclear RNA after cellular fractionation of tissue samples. In comparison with conventional polyA+ RNA, the cytoplasmic RNA contains a significantly higher fraction of exonic sequence, providing increased sensitivity in expression analysis and splice junction detection, and in improved *de novo* assembly of RNA-seq data. Conversely, the nuclear fraction shows an enrichment of unprocessed RNA compared with total RNA-seq, making it suitable for analysis of nascent transcripts and RNA processing dynamics.

**Conclusion:**

Our results show that cellular fractionation is a more rapid and cost effective approach than conventional polyA+ enrichment when studying mature RNAs. Thus, RNA-seq of separated cytosolic and nuclear RNA can significantly improve the analysis of complex transcriptomes from mammalian tissues.

## Background

The transcriptome is the complete catalogue of transcripts in the human cell. At any given time, a wide range of different RNA molecules are present at different levels of maturation and processing. The rates and the dynamics of RNA transcription and processing are unique for each cell. Previous studies have highlighted the importance of creating a complete map of transcripts, and the importance of understanding how different physiological conditions, developmental stages and disease can affect expression and regulation [1,2].

In the recent years, RNA-sequencing (RNA-seq) has emerged as a standard procedure to study transcriptomes and measure levels of gene expression [3-6]. Studies using this method have significantly expanded our understanding of transcriptome complexity and provided new insights into the mechanisms of gene expression and transcriptional regulation in development and disease [7-10]. However, many challenges still remain, primarily linked to data analysis and sample preparation [3,11].

The starting material for RNA-seq is typically total RNA or polyadenylated (polyA+) RNA, which both represent heterogeneous pools of RNA molecules at different stages of maturation and processing [12]. Several limitations arise from analyzing these populations of RNAs from whole cells. A commonly neglected problem is that total RNA, but also polyA+ RNA to a lesser extent, contains substantial amounts of intronic RNA originating from immature transcripts [13]. This intronic background coverage is accentuated in long genes expressed at high levels, and is most noticeable in brain tissue where long neuronal genes tend to be highly expressed. The presence of intronic RNAs may influence the sensitivity to detect transcripts, identify splice junctions and measure gene expression levels, as a large proportion of sequence reads is mapped to introns [14]. Also, oligo-dT purification steps are likely to introduce certain biases, such as unspecific retrieval of RNA containing poly-A stretches within the transcribed sequence, 5′ to 3′ biases, or truncated transcripts resulting either from alternative polyadenylation signals within introns or RNA degradation products [15-19].

Although total RNA-seq has been shown to provide insight into ongoing transcription and co-transcriptional splicing in the nucleus [14,20], the simultaneous presence of mature RNAs from the cytoplasm confounds the analysis of nuclear RNA maturation steps. Recently, two technologies, the genome-wide nuclear run-on sequencing (GRO-seq) and native elongating transcript sequencing (NET-seq), have been described to study nascent transcripts. GRO-seq yields an overview of transcription dynamics and directionality by labeling transcriptionally engaged nascent transcripts genome-wide, followed by high-throughput sequencing. NET-seq uses the stability of the ternary complex of DNA, RNA polymerase (RNAPII) and nascent RNA to capture and sequence nascent transcripts in living cells using endogenously expressed RNAPII with a 3 × −Flag epitope. These methods have successfully provided snapshots of ongoing transcription in cell lines [21,22]. However, these methods do not provide any insight into posttranscriptional events, and are based upon manipulation of the normal physiological conditions of the cells and require extensive optimization and standardization.

Recent improvements in RNA extraction protocols now make it possible to study specific pools of RNA molecules, either by fractionation of subcellular compartments or by molecular capture of specific RNA-associated targets. Several kits for RNA extraction from different cellular fractions are commercially available (e.g. Qiagen, Invitrogen and ThermoScientificBio). However, these kits are associated with significant amounts of cross contamination between the fractions. To overcome the effects of cross contamination, recent studies used the selection of polyA+ from the cytoplasmic fraction and chromatin-associated transcripts from the nuclear fraction to obtain more homogenous pools of mature and nascent transcripts respectively [20,23]. Although this represents an efficient approach, these protocols are time consuming and require high amounts of starting material. They are therefore less suitable for studies based on tissue samples, where starting material is often a limiting factor.

In this study, we investigate the benefits of analyzing RNA sequencing data from separated nuclear and cytosolic RNA. We have made improvements to an existing protocol for cell fractionation in order to more efficiently fractionate cytoplasmic and nuclear RNAs from tissue samples. We find that extraction of RNA using our modified protocol results in pure subcellular RNA populations with minimal levels of cross contamination. RNA-seq results from nuclear and cytosolic fractions are compared to polyA+ and total RNA-seq from the same tissue samples. Our results highlight significant advantages of performing RNA-seq on cytosol and nuclear RNAs, as compared to standard RNA-seq protocols. Sequencing of nuclear RNA provides insight into nascent transcript formation and processing, and cytosolic RNA-seq leads to improved de novo assembly and splice junction detection.

## Results

### Cellular fractionation of cytoplasm and nucleus

**Figure 1:**
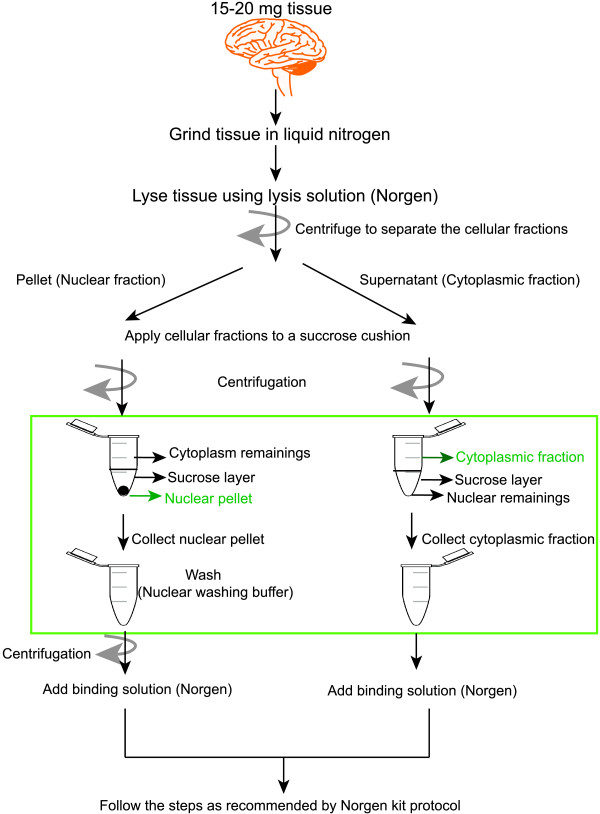
**Schematic representation of the extraction of cytoplasmic and nuclear RNA.** The modification steps introduced to the cytoplasmic and nuclear purification kit from Norgen are shown within the green rectangle. Locations of the cytoplasmic and nuclear fractions in the sucrose gradient are marked in green.

To improve the efficiency of RNA extraction from different subcellular fractions, the cytoplasmic and nuclear RNA purification kit (Norgen) was modified with the addition of a sucrose gradient and extra washing step (see Methods for more details). The method for cellular fractionation of RNA is outlined in Figure 1. Our results show that the nuclear RNA fractions are virtually free of ribosomal RNA and that the cytoplasmic RNA contained no traces of genomic DNA (Additional file 1: Figure S1). The extraction takes less than 1.5 hours and as little as 15 mg of tissue sample can be used as starting material, with no requirement for additional polyA+ or chromatin purification kits. In comparison, polyA+ purification requires a larger amount of starting material (50 mg) and takes a longer time to complete.

### Cytoplasmic and nuclear RNA-seq

**Figure 2:**
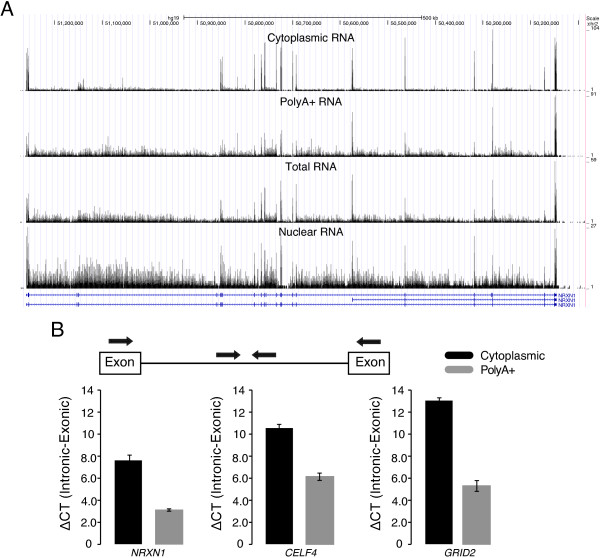
**Intronic read coverage for different RNA fractions. A)** RNA-seq coverage for Sample 2 across the gene *NRXN1* for the four different RNA fractions, viewed in the UCSC genome browser [24]. Long neuronal genes tend to yield a high coverage in introns in conventional RNA-seq data. The figure shows that fractions except the cytoplasmic RNA show a high coverage across the entire transcript, including the introns. **B)** Quantitative real-time PCR (qRT-PCR) quantification of intronic and exonic expression levels in cytoplasmic and polyA+ RNA. Primers were designed within an intron and the two surrounding exons for three genes (*NRXN1*, *CELF4* and *GRID2*) according to the schematic representation at the top (see Additional file 1: Table S2 for primer sequences). In all three cases, ΔCT (Intronic-Exonic) is higher in the cytoplasmic RNA compared to polyA+ RNA, while the opposite for intronic regions.

To investigate the separation and detection of mature transcripts from the cytoplasm and nascent transcripts from the nucleus, we purified cytoplasmic and nuclear RNA from two human fetal frontal cortex tissue samples, denoted Sample 1 and Sample 2. We then sequenced (SOLiD5500) the cytoplasmic and nuclear RNA from both samples, along with total and polyA+ RNA from the same tissues. While many genes have very clean peaks corresponding to the exons (Additional file 1: Figure S2), we find that long genes with high expression levels show a surprisingly high intron read coverage in nuclear, total and polyA+ RNA. Conversely, the RNA-seq coverage profiles revealed a striking enrichment of exonic reads in the cytoplasmic fraction compared to the other RNA fractions (see Figure 2A and Additional file 1: Figure S3). These differences in intronic and exonic RNA levels were validated using qRT-PCR by calculating amplification cycle number differences (ΔCT) between introns and exons in the cytoplasmic and polyA+ RNA fractions (Figure 2B). We used the raw (ΔCT) for calculations, without normalization, because house-keeping genes may not be equally represented in subcellular fractions as compared to total or polyA+ selected RNA. However, similar results were obtained when normalizing against beta-actin (Additional file 1: Figure S4). In order to show the effect of the extra steps added to the commercial RNA extraction protocol we also performed the same experiment on RNA extracted using the kit without modifications (Additional file 1: Figure S5).

In all experimentally validated genes, we found a higher ratio of exonic to intronic RNA in the cytoplasmic RNA compared with the polyA+ fraction, demonstrating the efficiency of our protocol to enrich for mature RNA transcripts (Figure 2B). Unexpectedly, polyA+ RNAs showed high levels of intron coverage across entire transcripts, contradicting the idea that polyA+ purification enriches exclusively for fully mature RNAs. To exclude issues with polyA+ purification as a reason for this, we sequenced a high quality adult brain polyA+ RNA acquired from a commercial vendor (Clontech). Since similar patterns were seen also for the commercial polyA+ sample, the observed intronic coverage is not likely to be an artifact unique to the polyA+ enrichment carried out in our laboratory. In line with these findings, recent studies indicate that transcripts may be polyadenylated prior to the completion of splicing [23,25]. This data, together with the biases associated with polyA+ selection, may potentially provide an explanation for the high level of intronic RNA in polyA+ data.

### Comparing exonic-to-intronic enrichment between RNA fractions

To quantify the relative enrichment of exonic reads compared to intronic on a global scale, we defined a ratio of exonic-to-intronic reads (denoted the *EI-ratio*). The *EI-ratio* is a number ranging from 1 (when all intragenic reads are exonic) to 0 (when all intragenic reads are intronic). In the cytoplasmic RNA, the *EI-ratios* were 0.74 and 0.72 in the two tissues. For polyA+ RNA the *EI-ratio* ranged from 0.27-0.45 and even lower values were seen in total RNA (0.20-0.42) and nuclear RNA (0.12-0.31) (see Table 1). As expected, these results show that intronic reads are present at high levels in the nuclear and total RNA, but also highlight that there is a substantial fraction of intronic reads in polyA+ RNA. Importantly, our results demonstrate that cytoplasmic RNA is significantly enriched for exons in comparison with all the other RNA populations, implying that it is a preferable extraction method for studying completely processed mRNA molecules. On the other hand, if the aim is to study nascent transcripts, our results suggest that nuclear RNA is the best choice since it gives the lowest *EI-ratios* (Table 1). Interestingly, we observed lower *EI-ratios* for the polyA+ and total RNA fractions in Sample 1 as compared to Sample 2. We explain this with biological differences in transcription levels between the two samples. Genes involved in the nervous system development often contain very long introns [26] and our results indicate that these genes in Sample 1, which is from an earlier developmental stage, are transcribed at much higher levels, resulting in a higher fraction of intronic reads.

**Table 1:** ***EI-ratios *** **for different RNA fractions**

	**Cytoplasmic**	**Nuclear**	**Total**	**PolyA+**
**Sample 1**	0.74	0.13	0.20	0.27
**Sample 2**	0.72	0.31	0.42	0.45
**Clontech**	N/A	N/A	N/A	0.33

### Cytoplasmic RNA-seq improves the analysis of mature mRNAs

**Figure 3:**
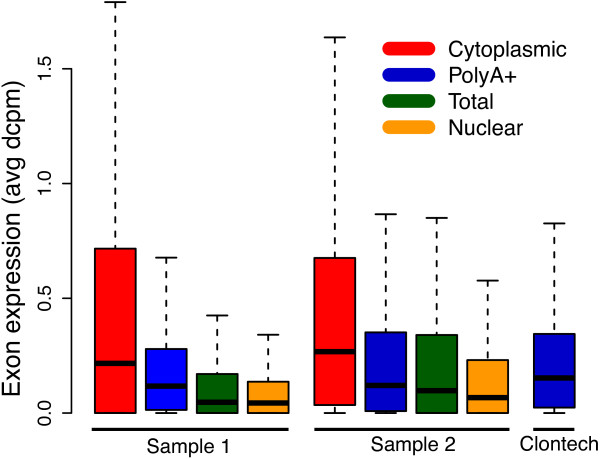
**Expression levels for different RNA fractions.** The figure shows expression levels of all human RefSeq exons, measured in average depth of coverage per million mapped reads (dcpm). Exons are more enriched in the cytoplasmic RNA compared to the other fractions. Compared to the nuclear RNA, the total RNA fraction showed 33% higher levels of exonic enrichment on average for the two samples. For polyA+ RNA the same percentage was 290% and for cytoplasmic RNA it was over 500%.

To further evaluate the different RNA fractions, we focused on the potential of our method to improve the detection and quantification of mature spliced transcripts. Given the higher *EI-ratio* in the cytoplasmic RNA, we expect that it should be possible to identify a larger number of mature transcripts in RNA-seq data from the cytoplasm compared with the other fractions. We first investigated the ability to detect expressed transcripts in the different RNA fractions, using the depth of coverage per million mapped reads (dcpm) as a measure to quantify the expression levels of all exons in the human genome. As expected, cytoplasmic RNA-seq gives the highest dcpm levels for exons (Figure 3). Furthermore, by analyzing dcpm values at exonic positions compared with the background noise signal represented by the coverage on the anti-sense strand (see Methods), we could estimate the number of expressed exons for each of the RNA fractions (see Additional file 1: Table S1). In the cytoplasmic RNA fraction we detect 8-19% more expressed exons than in polyA+ RNA and 29-49% more than in total RNA, thereby corroborating a more efficient detection of exonic reads in the cytoplasmic fraction as compared to the polyA+ or total RNA fractions.

We used TopHat [27] to perform splice junction analysis on RNA-seq data from sample 1. After correcting for differences in number of total sequence reads, the cytosolic RNA-seq were found to have around 10,000 (10.3%) more junctions than polyA+ RNA-seq (102,528 vs 92,951 junctions). Furthermore, the number of reads spanning splice junctions in the cytosolic RNA-seq were roughly 500,000 (34.9%) higher compared with the polyA+ RNA-seq (2,230,161 vs 1,653,225 junction reads). These results confirm that the number of reads derived from spliced transcripts is greater in the cytosolic RNA, and that each junction detected has better support in the cytosolic RNA-seq data.

To evaluate the data in an unbiased way, without any prior information about gene coordinates, we then performed a *de novo* transcriptome assembly for each RNA population using the Trinity software [28]. Here, we analyzed Sample 2 since it represents a more challenging dataset with smaller differences in *EI-ratios* between RNA fractions. The results show that the cytoplasmic RNA fraction provides longer contigs, and featured 30% more transcripts longer than 1 kb compared with the polyA+ fraction and almost 10 times more than in total RNA (see Table 2). This trend is consistent using a cutoff of 2 kb. There were also more transcripts containing open reading frames (ORFs) in the cytoplasmic fraction. Despite of the fact that our RNA-seq data consists of short (75 bp) and unpaired reads, which are not ideal for *de novo* assembly, our results clearly show that cytoplasmic RNA gives a better transcriptome assembly compared with the other fractions.

**Table 2:** Comparison between expression and transcript assemblies for sample 2

	**Cytoplasmic**	**PolyA+**	**Total**	**Nuclear**
Number expressed exons	241704	203924	187836	168962
N50 size	428	354	308	-
Bases in transcripts > 500 nt (M)	14.7	13.3	1.9	-
Bases in transcripts > 1000 nt (M)	4.5	3.5	0.5	-
ORFs > 100aa	6,323	6,028	883	-

Shown are the N50 sizes (half of the bases in the assembly resides in contigs this size or larger), the total bases contained in contigs larger than 500 and 1000 nucleotides and the number of transcripts with open reading frames (ORF) larger than 100 amino acids. In all cases, the cytoplasmic assembly outperforms polyA+ selection, as well as Total RNA. Trinity could not assemble the nuclear fraction due to long run times and we terminated the process after taking up 2.5 CPU years.

### Nuclear RNA-seq improves analysis of nascent transcription

**Figure 4:**
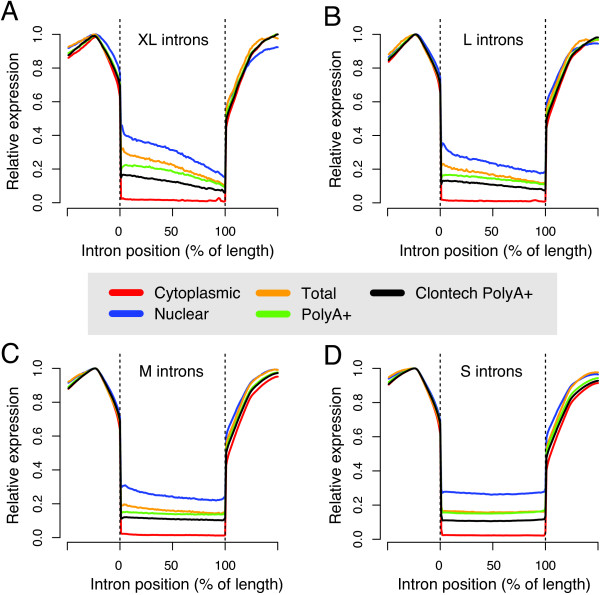
**RNA-seq coverage profiles across introns of different sizes.** The average RNA-seq coverage was computed for all different RNA-seq fractions of Sample 1 and the polyA+ RNA sample acquired from Clontech, by dividing each intron into 100 bins. The regions flanking the introns (to the left and right of the dotted lines, respectively) show the average coverage in 50 bp regions upstream and downstream of the introns. The panels show the average coverage profiles for **A)** all 1567 ‘XL’ introns of size at least 100 kb. **B)** 2792 ‘L’ introns of size 50–100 kb. **C)** 19996 ‘M’ introns of size 10–50 kb **D)** 98636 ‘S’ introns of size 1–10 kb.

The low EI-ratios in the nuclear RNA fractions suggest that a high amount of nascent transcripts are being detected by nuclear RNA sequencing. To investigate this further we performed a global analysis of the sequence coverage across introns. Figure 4 shows the coverage for all four RNA fractions and the commercial polyA+ sample across introns of different lengths. It has previously been observed that nascent transcripts give rise to a 5′-3′ negative gradient of RNA-seq coverage across long introns [14], and consistent with this we see such slopes in global analyses of long introns for the nuclear, total and polyA+ fractions (see Figure 4A-B). The 5′-3′ slope is associated with nascent transcript production and this pattern can also be used as an indicator of splicing dynamics [14,29,30]. The steepest slopes are detected for the nuclear RNA, indicating that it is the RNA fraction containing the highest amount of nascent transcripts. The second steepest slopes are found in total RNA, followed by polyA+ RNA. In contrast, the intronic coverage in the cytoplasmic RNA-seq fraction is almost negligible and no slope at all is seen in the cytoplasmic RNA. For shorter introns the 5′-3′ gradients become less evident (see Figure 4C-D), and likely the reason for this is that the RNA polymerase moves too quickly through the small introns to generate a detectable gradient of nascent RNA. Our results thus show that the nuclear fraction contains the highest amount of nascent RNAs, providing a more distinct 5′-3′ slope (see Figure 4), indicating that sequencing of nuclear RNA is preferable over total RNA for studying ongoing transcription.

## Discussion

The advent of RNA-Seq has for the first time provided a method to examine the RNA in cells and tissues in an unbiased way. However, the methods of extracting RNA differs and may give very different starting material for the RNA-Seq experiment. It is important to understand the advantages and disadvantages of different extraction protocols, and the biases that are introduced in the RNA preparation experiments. There are now several RNA protocols available for extracting specific classes of RNA from the cell prior to sequencing. These protocols are based on separation by size (e.g. microRNA), type of RNA or association with certain subcellular compartments or structures. Examples include polyA+ selection, extraction of chromatin associated RNA and extraction of cytosolic RNA [20,23,31] and RNA polymerase associated RNA [22]. Most data presented for subcellular fractions of RNA are based on cell lines [20,23]. Here we present the improvement of a method for separation of cytosolic and nuclear RNA and demonstrate that for many applications this is a more informative approach than sequencing polyA+ and total RNA.

In addition to the improved efficiency of this method to enrich for mature RNA transcripts, cytoplasmic RNA purification provides several technical advantages over conventional polyA+ enrichment approaches. The enrichment of polyA+ RNA directly from tissues and cell lines is a complicated and time-consuming procedure, requiring significant amounts of starting material. Cytoplasmic RNA purification represents a more affordable and rapid protocol (1.5 hours). Moreover, it requires only 15 mg of tissue to provide sufficient amounts of RNA from both cytosol and nuclear fractions for RNA-seq and subsequent validation experiments. Our approach has lower resolution for specific sub-classes of RNA compared to more specialized molecular approaches such as GRO-seq and NET-seq, but has the benefit of providing a global picture of all RNA in the cell, with increased resolution compared with conventional extraction protocols.

There are several potential biases when using polyA+ RNA, which may be avoided using the cytosolic fraction. We find a surprisingly high fraction of intronic sequence reads in the polyA+ RNA-Seq data. Although the results were better for the polyA+ RNA purchased from a commercial vendor, where a more stringent selection protocol was applied, we still find that > 50% of the intragenic reads map to introns. These findings indicate that there may be significant unspecific capture of RNA in the polyA+ selection step, irrespective of the protocol used. Another explanation for the high intronic background in polyA+ data is that some fraction of introns (or transcripts) is spliced only after the addition of the polyA-tail [23]. Such a mechanism would explain why certain genes give a high intronic background in sequencing of polyA-selected RNA. The coverage profiles across introns are clearly visible in Figure 4, where it is obvious that the polyA+ baseline read coverage is significantly higher than in the RNA extracted from the cytosol. There have also been reports indicating that selection of mononucleotide stretches of adenines within nascent RNA may create noise in polyA+ seq data. Many of these biases are avoided by extracting only on the mature transcripts present in the cytosol. The cytosolic RNA contains high amounts of rRNA that needs to be depleted prior to sequencing. There are reports that rRNA depletion may introduce 5′-3′ bias across transcripts [32]. However, published data suggest that polyA+ selected data show more 5′-3′ bias than does ribo-minus RNA-seq data [32,33]. If other biases are introduced by rRNA depletion, biases in cytosolic RNA-seq data would be similar to those in conventional total RNA-seq, which also requires rRNA depletion prior to sequencing.

Compared to cytosolic RNA, the nuclear fraction is less well suited for measuring mRNA expression levels. Instead, the enrichment of nascent RNA from the nuclear fraction is suitable for studying transcription dynamics without any further rRNA depletion steps. We show that signatures of co-transcriptional splicing are more distinct in the nuclear fraction than in total RNA. The level of intron coverage is also a good indicator of the rate of nascent transcript production, which does not always correlate with the level of processed mRNA. Additionally, nuclear extraction is advantageous for studies of RNA molecules that are primarily present in the nucleus, such as pri-microRNA, rRNA precursors and some long non-coding RNAs [34-36].

By extracting both cytosolic and nuclear RNA fractions from the same tissue, it is also possible to study the relative abundance of the same transcripts in each fraction. Such analysis may provide insight into transcript processing, turnover and degradation. For example, a high level of nascent transcription for a transcript found at relatively lower levels in the cytosol might be an indication of rapid cytosol turnover. Conversely, high levels a transcript in the cytosolic RNA, combined with low nuclear levels for the same transcript may be an indication of high stability and long half-life for the mRNA of that transcript.

## Conclusion

In this paper, we report on the advantages of using RNA-seq on separated cytosolic and nuclear RNA, extracted using a modified and improved protocol. Analysis on separated nuclear and cytoplasmic fractions is valuable to study RNA degradation patterns (degradome) and transport dynamics, intron retention patterns and mRNA turnover. Our results show that extraction of nuclear RNA is better than total RNA for measuring of nascent transcript levels and for studies of mechanisms of splicing. Furthermore, we show that RNA-sequencing of the cytoplasmic fraction shows an increased exonic coverage and minimal levels of intronic reads. This results in significantly higher number of transcripts that can be assembled from this fraction when compared to total or polyA+ preparations. Our data shows that sequencing of cytosolic RNA yields substantially lower background from immature transcripts, and we propose that cytosolic RNA-seq should be the method of choice for *de novo* transcriptome assembly and tissue specific expression profiling.

## Methods

### Samples

Tissue and total RNA samples from two fetal frontal cortex tissues, 23/24 weeks female (Sample 1) and 38 weeks male (Sample 2) were purchased from Capital Biosciences. The commercial adult frontal lobe polyA+ RNA sample with two rounds of polyA+ selection was acquired from Clontech (Catalogue number: 636165).

### Cytoplasmic and nuclear RNA extraction

Cytoplasmic and nuclear RNA was purified from two fetal frontal cortex using Cytoplasmic and nuclear RNA purification kit (Norgen) with modifications as illustrated in Figure 1. In short, 20 mg of frozen tissue were grinded in liquid nitrogen using mortar and pestle. Tissue powder was transferred to ice cold 1.5 ml tubes. Then, 200 μl lysis buffer (Norgen) was added to the grinded tissue. The tubes were incubated on ice for 10 minutes and then centrifuged for 3 minutes at 13,000 RPM to separate the cellular fractions.

The supernatant containing the cytoplasmic fraction and the pellet containing the nuclei were mixed with 400 μl 1.6 M sucrose solution and carefully layered on the top of two 500 μl sucrose solution in two separate tubes. Both fractions were the centrifuged on 13,000-RPM for 15 minutes (4C°). The cytoplasmic fraction was collected form the top of the sucrose cushion and the cytoplasmic RNA was then further purified according to Norgen kit recommendations. The nuclear pellet was collected from the bottom of the tube and washed with 200 μl 1× PBS. The nuclei were collected after another centrifugation at 13,000 RPM for 3 minutes. The nuclear RNA was purified from the nuclear fraction according to the Norgen kit recommendations.

### polyA+ RNA purification

polyA+ RNA from sample 1 and 2 was purified from 1 μg total RNA using MicroPoly(A)Purist kit (Ambion) according to the manufacturer’s instructions.

### Preparation of cDNA and quantitative real time PCR (qrtPCR)

Starting with 1 μg of cytoplasmic or nuclear RNA, cDNA was synthesized using the Maxima first strand cDNA synthesis kit (Fermentas) according to the manufacturer’s recommendations. 1 μl of the resulting cDNA was used for qrtPCR to measure the relative intronic and exonic expression in each fraction. The qrtPCR was performed with Stratagene Mx3000P in 96-well plates. The reactions were carried out with an initial denaturation at 95°C for 10 min followed by 40 cycles of denaturation at 95°C for 15 s, primer annealing at 60°C for 30 s and extension at 72°C for 30 s.

The qrtPCR contained 12.5 ng single stranded cDNA, 0.4 μM for each primer and 12.5 μl Maxima SYBR Green/ROX qPCR Master Mix (Fermentas) in 25 μl reactions. All samples were amplified in triplicate and the mean values were used to calculate the expression level of each target. The intronic/exonic expression level ratios were determined by calculating the differences between the CT values (ΔCT) for exonic and intronic expression for each gene in each fraction. Raw data were analyzed using MxPro Mx3000P software (Stratagene).

### Preparation of RNA-seq libraries from cytoplasmic and total RNA

The quality of the input RNA was controlled using a RNA 6000 Pico chip on a Bioanalyzer (Agilent Technologies) and only RIN-values above 7 were accepted. Removal of rRNA was performed using the RiboMinus Eukaryote Kit (Life Technologies) according to manufacturer’s protocols. The samples were then fragmented using RNaseIII for 7 min. RNA libraries were constructed using the AB Library Builder Whole Transcriptome Core Kit (Life Technologies) and amplified (12 cycles). Emulsion PCR was performed using the EZ Bead System (Life Technologies).

### Preparation of RNA-seq libraries from nuclear and polyA+ RNA

polyA+ and nuclear RNA samples were checked for rRNA contamination using a RNA 6000 Pico chip on a Bioanalyzer (Agilent Technologies). The samples were then fragmented using RNaseIII for 5 min. RNA libraries were constructed using the AB Library Builder Whole Transcriptome Core Kit (Life Technologies) and amplified 15 cycles. Emulsion PCR was performed using the EZ Bead System (Life Technologies).

### RNA sequencing and alignment of reads

The RNA-seq libraries were sequenced on the SOLiD5500xl system, generating reads of length 75 bp. All four RNA fractions from Sample 2 were sequenced using the Exact Call Chemistry (ECC), which enables high accuracy conversion of reads from ‘color space’ to normal nucleotide sequences. This was done to facilitate the comparative *de novo* transcriptome assembly analysis of RNA fractions from Sample 2. Reads for all samples were aligned to the human reference genome (hg19 assembly) using v2.5 of the LifeScope software.

### Detection of significantly expressed exons

The expression level for each exon was quantified from the RNA-seq data using the average depth of coverage per million mapped reads (average dcpm) as proposed by Hillier et al. [37]. The dcpm values are comparable between all RNA-seq datasets, since it normalizes for differences in mapping efficiency and number of reads generated. To calculate the number of expressed exons in each sample, we established a cut-off threshold based on the dcpm values at exonic positions on the opposite (anti-sense) strand. The coverage on the anti-sense strand largely represent the background noise in the experiment, and the dcpm cut-off was set at the 99th percentile, i.e. so that 99% of the exonic positions on the anti-sense strand were below the threshold. Two different ways to calculate the cut-off were tried. The first approach was based on all samples put together, giving an identical dcpm cut-off value for all samples. Alternatively, we calculated separate dcpm cut-offs for each sample. For both methods we then recorded the number of exons with expression levels above the thresholds. The cut-off levels and number of expressed exons in each sample are presented in Additional file 1: Table S1.

### Splice junction detection

TopHat 2.0.8b [27] and Bowtie 1.0.0 [38] were used to detect splice junction in the cytosolic and polyA+ RNA-seq data in sample 1. To correct for differences in read counts between the two RNA fractions, random reads were drawn from the cytosolic RNA sequencing to obtain equal number of reads between the two datasets. The programs were run using the standard settings for colorspace as recommended in the manual.

### Calculating EI-ratios

To calculate the Exon-Intron ratios (*EI-ratios*), the BEDTools software [39] was used to extract the number of reads overlapping with exons (*E*) and introns (*I*). Only reads mapping to the same strand as the gene were considered for this analysis. Having extracted the exonic and intronic reads, the *EI-ratio* was defined as *E*/(*E + I*).

### De novo assembly of RNA-seq reads

Since the sequenced libraries yielded different numbers of reads, we randomly down-sampled the reads for the cytosolic fraction and total RNA to be the same in all cases (60,726,591). We note that down-sampling might introduce bias, but if so, then in favor of the polyA+ selected sample, which had the lowest read counts. We then ran *Trinity* on each data set with default parameters. For evaluation and comparison of open reading frames, we required a start codon and stop codon to be part of a transcript. For comparing transcripts to the Ensembl gene build, we used blat [40] requiring a minimum alignment length of 100 nucleotides, and identity > 98%.

### Accession codes

RNA-Seq reads are available in the ArrayExpress database (http://www.ebi.ac.uk/arrayexpress) under accession number E-MTAB-1898.

## Competing interests

The authors declare that they have no competing interests.

## Authors’ contributions

AZ, LC and LF conceived and designed the study. AA, AZ, LC and LF planned and coordinated experiments and analysis. AZ performed the sample preparation and experimental analysis. LN performed the sequencing library preparations. AA and JH performed alignments, splice junction detection, gene based analysis and statistical analyses. MG was responsible for de novo assembly. All authors participated in discussions of different parts of the study. AA, AZ, LC and LF wrote the manuscript. All authors read and approved the manuscript.

## Supplementary Material

Additional file 1 **The following additional data are available with the online version of this paper.** Additional data file 1 is an assessment of the cytoplasmic and nuclear RNA purification using Norgen kit only or with modification. Additional data file 2 is a figure illustrating RNA-seq coverage for *CELF4* and *GRID2* from sample 1, viewed in UCSC genome browser. Additional data file 3 is a figure showing the raw data (CT values) differences between cytoplasmic and polyA+ selected RNA populations. Additional data file 4 is a table listing the cut-off values and number of expressed exons out of refSeq exons. Additional data file 5 is a table listing primer sequences for the quantification of intronic and exonic expression in *NRXN1, CELF4* and *GRID2.* Click here for file

## References

[B1] Wang GS Cooper TA Splicing in disease: disruption of the splicing code and the decoding machinery Nat Rev Genet 2007 8 10 749 761 10.1038/nrg2164 17726481

[B2] Nilsen TW Graveley BR Expansion of the eukaryotic proteome by alternative splicing Nature 2010 463 7280 457 463 10.1038/nature08909 20110989 PMC3443858

[B3] Mortazavi A Williams BA McCue K Schaeffer L Wold B Mapping and quantifying mammalian transcriptomes by RNA-Seq Nat Methods 2008 5 7 621 628 10.1038/nmeth.1226 18516045 PMC13303166

[B4] Wilhelm BT Marguerat S Watt S Schubert F Wood V Goodhead I Penkett CJ Rogers J Bahler J Dynamic repertoire of a eukaryotic transcriptome surveyed at single-nucleotide resolution Nature 2008 453 7199 1239 1243 10.1038/nature07002 18488015

[B5] Marioni JC Mason CE Mane SM Stephens M Gilad Y RNA-seq: an assessment of technical reproducibility and comparison with gene expression arrays Genome Res 2008 18 9 1509 1517 10.1101/gr.079558.108 18550803 PMC2527709

[B6] Wu JQ Du J Rozowsky J Zhang Z Urban AE Euskirchen G Weissman S Gerstein M Snyder M Systematic analysis of transcribed loci in ENCODE regions using RACE sequencing reveals extensive transcription in the human genome Genome Biol 2008 9 1 R3 10.1186/gb-2008-9-1-r3 18173853 PMC2395237

[B7] Sultan M Schulz MH Richard H Magen A Klingenhoff A Scherf M Seifert M Borodina T Soldatov A Parkhomchuk D A global view of gene activity and alternative splicing by deep sequencing of the human transcriptome Science 2008 321 5891 956 960 10.1126/science.1160342 18599741

[B8] Wang Z Gerstein M Snyder M RNA-Seq: a revolutionary tool for transcriptomics Nat Rev Genet 2009 10 1 57 63 10.1038/nrg2484 19015660 PMC2949280

[B9] Pandit S Zhou Y Shiue L Coutinho-Mansfield G Li H Qiu J Huang J Yeo GW Ares M Jr Fu XD Genome-wide analysis reveals sr protein cooperation and competition in regulated splicing Mol Cell 2013 50 2 223 235 10.1016/j.molcel.2013.03.001 23562324 PMC3640356

[B10] Halvardson J Zaghlool A Feuk L Exome RNA sequencing reveals rare and novel alternative transcripts Nucleic Acids Res 2013 41 1 e6 10.1093/nar/gks816 22941640 PMC3592422

[B11] Ramskold D Wang ET Burge CB Sandberg R An abundance of ubiquitously expressed genes revealed by tissue transcriptome sequence data PLoS Comput Biol 2009 5 12 e1000598 10.1371/journal.pcbi.1000598 20011106 PMC2781110

[B12] van Bakel H Nislow C Blencowe BJ Hughes TR Most “dark matter” transcripts are associated with known genes PLoS Biol 2010 8 5 e1000371 10.1371/journal.pbio.1000371 20502517 PMC2872640

[B13] Wetterbom A Ameur A Feuk L Gyllensten U Cavelier L Identification of novel exons and transcribed regions by chimpanzee transcriptome sequencing Genome Biol 2010 11 7 R78 10.1186/gb-2010-11-7-r78 20653958 PMC2926789

[B14] Ameur A Zaghlool A Halvardson J Wetterbom A Gyllensten U Cavelier L Feuk L Total RNA sequencing reveals nascent transcription and widespread co-transcriptional splicing in the human brain Nat Struct Mol Biol 2011 18 12 1435 1440 10.1038/nsmb.2143 22056773

[B15] Gibbons JG Janson EM Hittinger CT Johnston M Abbot P Rokas A Benchmarking next-generation transcriptome sequencing for functional and evolutionary genomics Mol Biol Evol 2009 26 12 2731 2744 10.1093/molbev/msp188 19706727

[B16] Levin JZ Yassour M Adiconis X Nusbaum C Thompson DA Friedman N Gnirke A Regev A Comprehensive comparative analysis of strand-specific RNA sequencing methods Nat Methods 2010 7 9 709 715 10.1038/nmeth.1491 20711195 PMC3005310

[B17] Nam DK Lee S Zhou G Cao X Wang C Clark T Chen J Rowley JD Wang SM Oligo(dT) primer generates a high frequency of truncated cDNAs through internal poly(A) priming during reverse transcription Proc Natl Acad Sci U S A 2002 99 9 6152 6156 10.1073/pnas.092140899 11972056 PMC122918

[B18] Nagalakshmi U Wang Z Waern K Shou C Raha D Gerstein M Snyder M The transcriptional landscape of the yeast genome defined by RNA sequencing Science 2008 320 5881 1344 1349 10.1126/science.1158441 18451266 PMC2951732

[B19] Shcherbik N Wang M Lapik YR Srivastava L Pestov DG Polyadenylation and degradation of incomplete RNA polymerase I transcripts in mammalian cells EMBO Rep 2010 11 2 106 111 10.1038/embor.2009.271 20062005 PMC2828747

[B20] Tilgner H Knowles DG Johnson R Davis CA Chakrabortty S Djebali S Curado J Snyder M Gingeras TR Guigo R Deep sequencing of subcellular RNA fractions shows splicing to be predominantly co-transcriptional in the human genome but inefficient for lncRNAs Genome Res 2012 22 9 1616 1625 10.1101/gr.134445.111 22955974 PMC3431479

[B21] Core LJ Waterfall JJ Lis JT Nascent RNA sequencing reveals widespread pausing and divergent initiation at human promoters Science 2008 322 5909 1845 1848 10.1126/science.1162228 19056941 PMC2833333

[B22] Churchman LS Weissman JS Nascent transcript sequencing visualizes transcription at nucleotide resolution Nature 2011 469 7330 368 373 10.1038/nature09652 21248844 PMC3880149

[B23] Bhatt DM Pandya-Jones A Tong AJ Barozzi I Lissner MM Natoli G Black DL Smale ST Transcript dynamics of proinflammatory genes revealed by sequence analysis of subcellular RNA fractions Cell 2012 150 2 279 290 10.1016/j.cell.2012.05.043 22817891 PMC3405548

[B24] Kent WJ Sugnet CW Furey TS Roskin KM Pringle TH Zahler AM Haussler D The human genome browser at UCSC Genome Res 2002 12 6 996 1006 12045153 10.1101/gr.229102 PMC186604

[B25] Brody Y Neufeld N Bieberstein N Causse SZ Bohnlein EM Neugebauer KM Darzacq X Shav-Tal Y The in vivo kinetics of RNA polymerase II elongation during co-transcriptional splicing PLoS Biol 2011 9 1 e1000573 10.1371/journal.pbio.1000573 21264352 PMC3019111

[B26] Polymenidou M Lagier-Tourenne C Hutt KR Huelga SC Moran J Liang TY Ling SC Sun E Wancewicz E Mazur C Long pre-mRNA depletion and RNA missplicing contribute to neuronal vulnerability from loss of TDP-43 Nat Neurosci 2011 14 4 459 468 10.1038/nn.2779 21358643 PMC3094729

[B27] Trapnell C Pachter L Salzberg SL TopHat: discovering splice junctions with RNA-Seq Bioinformatics 2009 25 9 1105 1111 10.1093/bioinformatics/btp120 19289445 PMC2672628

[B28] Grabherr MG Haas BJ Yassour M Levin JZ Thompson DA Amit I Adiconis X Fan L Raychowdhury R Zeng Q Full-length transcriptome assembly from RNA-Seq data without a reference genome Nat Biotechnol 2011 29 7 644 652 10.1038/nbt.1883 21572440 PMC3571712

[B29] Carrillo Oesterreich F Preibisch S Neugebauer KM Global analysis of nascent RNA reveals transcriptional pausing in terminal exons Mol Cell 2010 40 4 571 581 10.1016/j.molcel.2010.11.004 21095587

[B30] Windhager L Bonfert T Burger K Ruzsics Z Krebs S Kaufmann S Malterer G L’Hernault A Schilhabel M Schreiber S Ultrashort and progressive 4sU-tagging reveals key characteristics of RNA processing at nucleotide resolution Genome Res 2012 22 10 2031 2042 10.1101/gr.131847.111 22539649 PMC3460197

[B31] Liao JY Ma LM Guo YH Zhang YC Zhou H Shao P Chen YQ Qu LH Deep sequencing of human nuclear and cytoplasmic small RNAs reveals an unexpectedly complex subcellular distribution of miRNAs and tRNA 3′ trailers PLoS One 2010 5 5 e10563 10.1371/journal.pone.0010563 20498841 PMC2871053

[B32] Tariq MA Kim HJ Jejelowo O Pourmand N Whole-transcriptome RNAseq analysis from minute amount of total RNA Nucleic Acids Res 2011 39 18 e120 10.1093/nar/gkr547 21737426 PMC3185437

[B33] Cui P Lin Q Ding F Xin C Gong W Zhang L Geng J Zhang B Yu X Yang J A comparison between ribo-minus RNA-sequencing and polyA-selected RNA-sequencing Genomics 2010 96 5 259 265 10.1016/j.ygeno.2010.07.010 20688152

[B34] Zhang Q Chen CY Yedavalli VS Jeang KT NEAT1 long noncoding RNA and paraspeckle bodies modulate HIV-1 posttranscriptional expression MBio 2013 4 1 00596 00512 10.1128/mBio.00596-12 PMC3560530 23362321

[B35] Rouquette J Choesmel V Gleizes PE Nuclear export and cytoplasmic processing of precursors to the 40S ribosomal subunits in mammalian cells EMBO J 2005 24 16 2862 2872 10.1038/sj.emboj.7600752 16037817 PMC1187937

[B36] Jeffries CD Fried HM Perkins DO Nuclear and cytoplasmic localization of neural stem cell microRNAs RNA 2011 17 4 675 686 10.1261/rna.2006511 21363885 PMC3062178

[B37] Hillier LW Reinke V Green P Hirst M Marra MA Waterston RH Massively parallel sequencing of the polyadenylated transcriptome of C. elegans Genome Res 2009 19 4 657 666 10.1101/gr.088112.108 19181841 PMC2665784

[B38] Langmead B Trapnell C Pop M Salzberg SL Ultrafast and memory-efficient alignment of short DNA sequences to the human genome Genome Biol 2009 10 3 R25 10.1186/gb-2009-10-3-r25 19261174 PMC2690996

[B39] Quinlan AR Hall IM BEDTools: a flexible suite of utilities for comparing genomic features Bioinformatics 2010 26 6 841 842 10.1093/bioinformatics/btq033 20110278 PMC2832824

[B40] Kent WJ BLAT–the BLAST-like alignment tool Genome Res 2002 12 4 656 664 11932250 10.1101/gr.229202 PMC187518

