# Supplementary material for: Efficient cellular fractionation improves RNA sequencing analysis of mature and nascent transcripts from human tissues
Source: BMC Biotechnol. 2013 Nov 13;13:99. doi: 10.1186/1472-6750-13-99 (PMC3833653; doi:10.1186/1472-6750-13-99)
Supplement: Additional file 1 — The following additional data are available with the online version of this paper. Additional data file 1 is an assessment of the cytoplasmic and nuclear RNA purification using Norgen kit only or with modification. Additional data file 2 is a figure illustrating RNA-seq coverage for CELF4 and GRID2 from sample 1, viewed in UCSC genome browser. Additional data file 3 is a figure showing the raw data (CT values) differences between cytoplasmic and polyA+ selected RNA populations. Additional data file 4 is a table listing the cut-off values and number of expressed exons out of refSeq exons. Additional data file 5 is a table listing primer sequences for the quantification of intronic and exonic expression in NRXN1, CELF4 and GRID2. [file 1472-6750-13-99-S1.doc]

**Supplementary Figures**


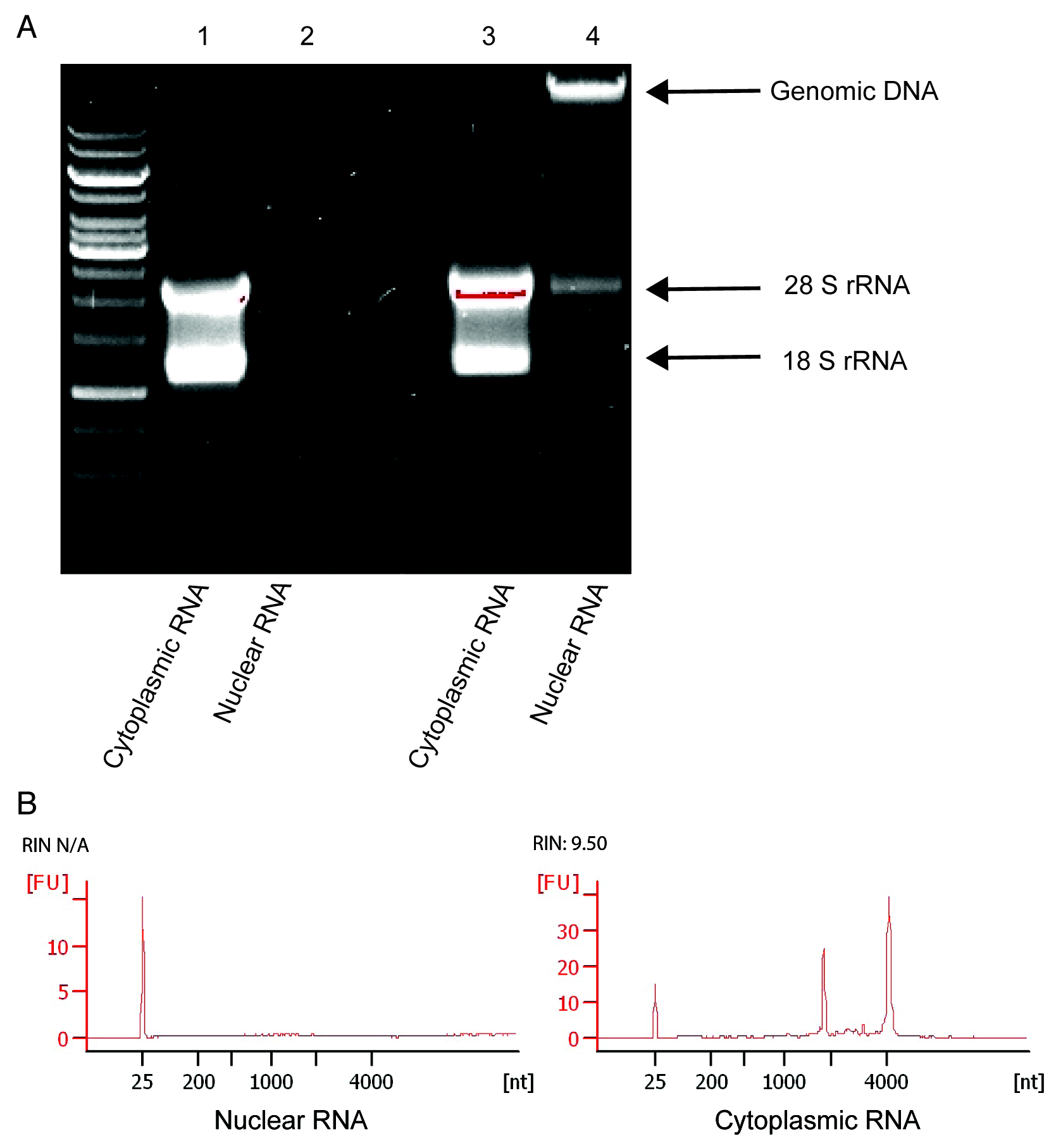


**Supplementary Figure S1.** Cytoplasmic and nuclear RNA purified from brain tissue using the Cytoplasmic and nuclear RNA purification kit (Norgen) with modifications. A) Agarose gel electrophoresis showing the quality of the purified RNAs. Samples 1 and 2 show results with modifications and samples 3 and 4 without modifications. The three bands in lane 4 represent the ribosomal RNA, reflecting the amount of cross- contamination between nucleus and cytoplasmic RNA fractions. These bands completely disappeared in well 2 (kit with modifications) indicating that the RNA purified using this protocol contains low cross-contamination between the RNA populations. B) High quality retrieval of Cytoplasmic and nuclear RNA was tested using the Bioanalyzer. The ribosomal RNA was restricted to the cytoplasmic RNA fraction.

**
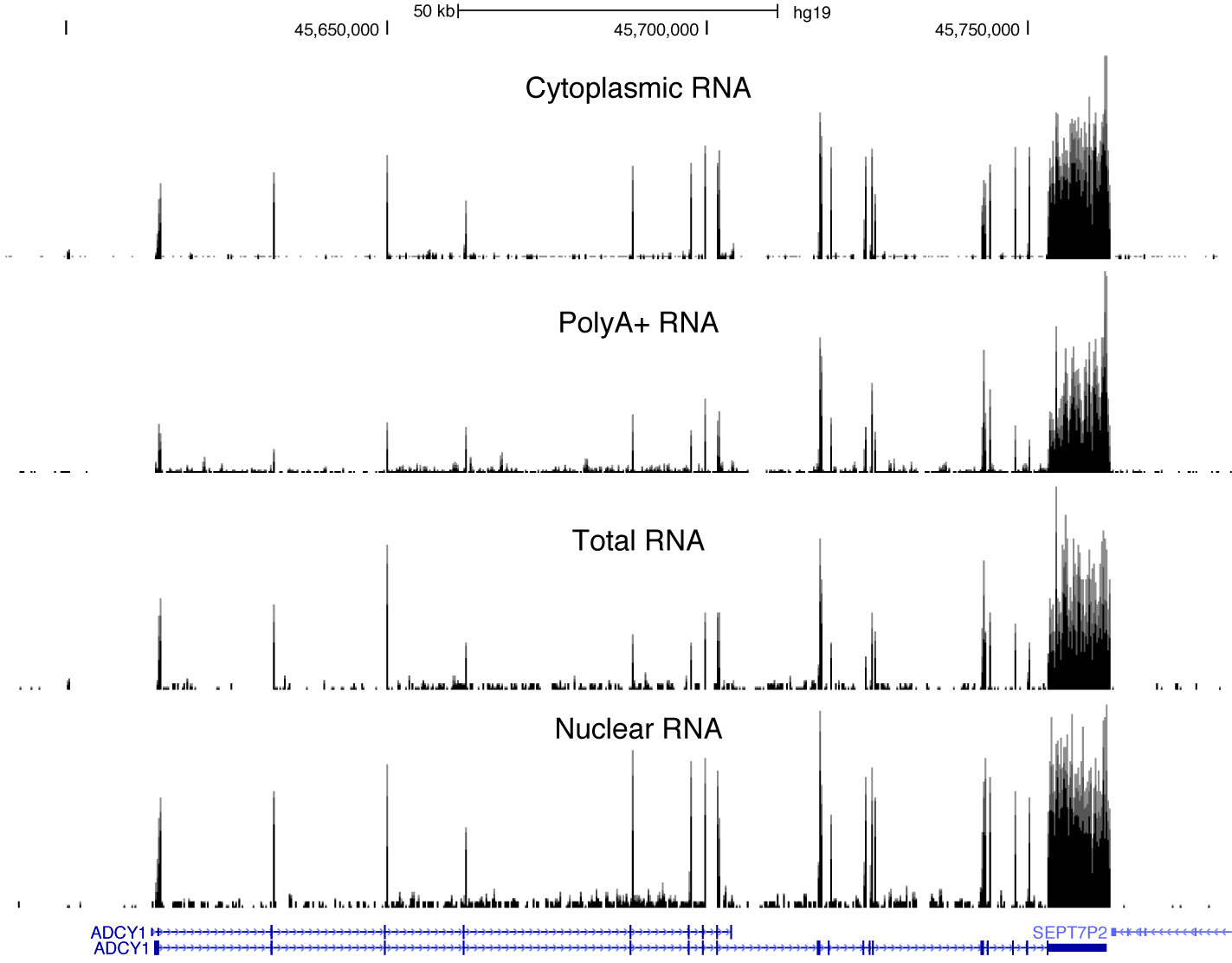
**

**Supplementary Figure S2.** RNA-seq coverage for *ACDY1*, viewed in the UCSC genome browser. Data is shown for all four RNA fractions of Sample 1. This is an example of a highly expressed gene showing lower abundance of intronic reads compared to *CELF4* and *GRID2* in supplementary Figure S3 below. The difference in coverage profiles can (at least partly) be explained by the fact that the *ACDY1* gene is much shorter than *CELF4* and *GRID2*, giving rise to a lower abundance of nascent RNAs.

**
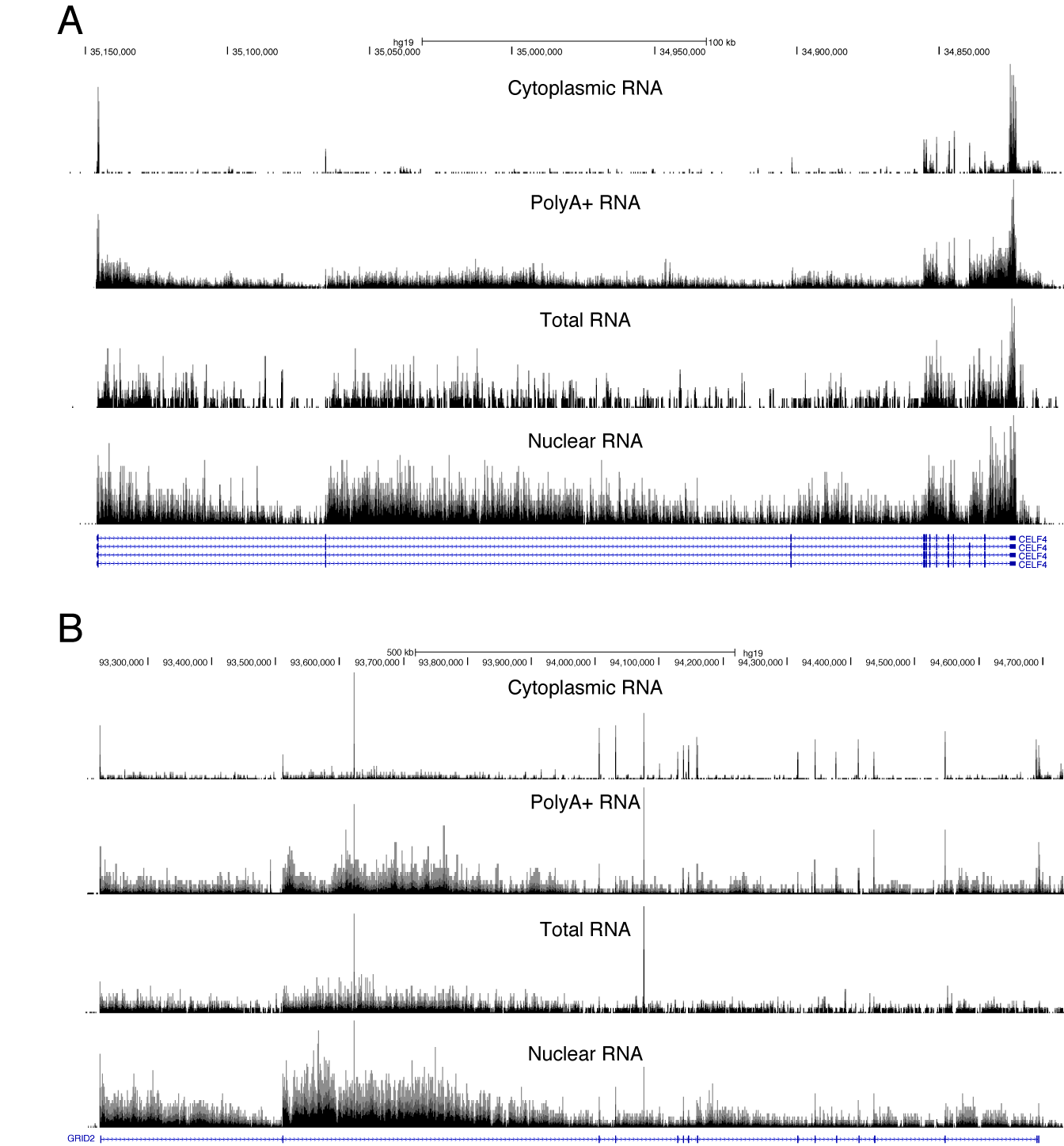
**

**Supplementary Figure S3.** RNA-seq coverage for *CELF4* and *GRID2*, viewed in the UCSC genome browser. Data is shown for all four RNA fractions of Sample 1.

**
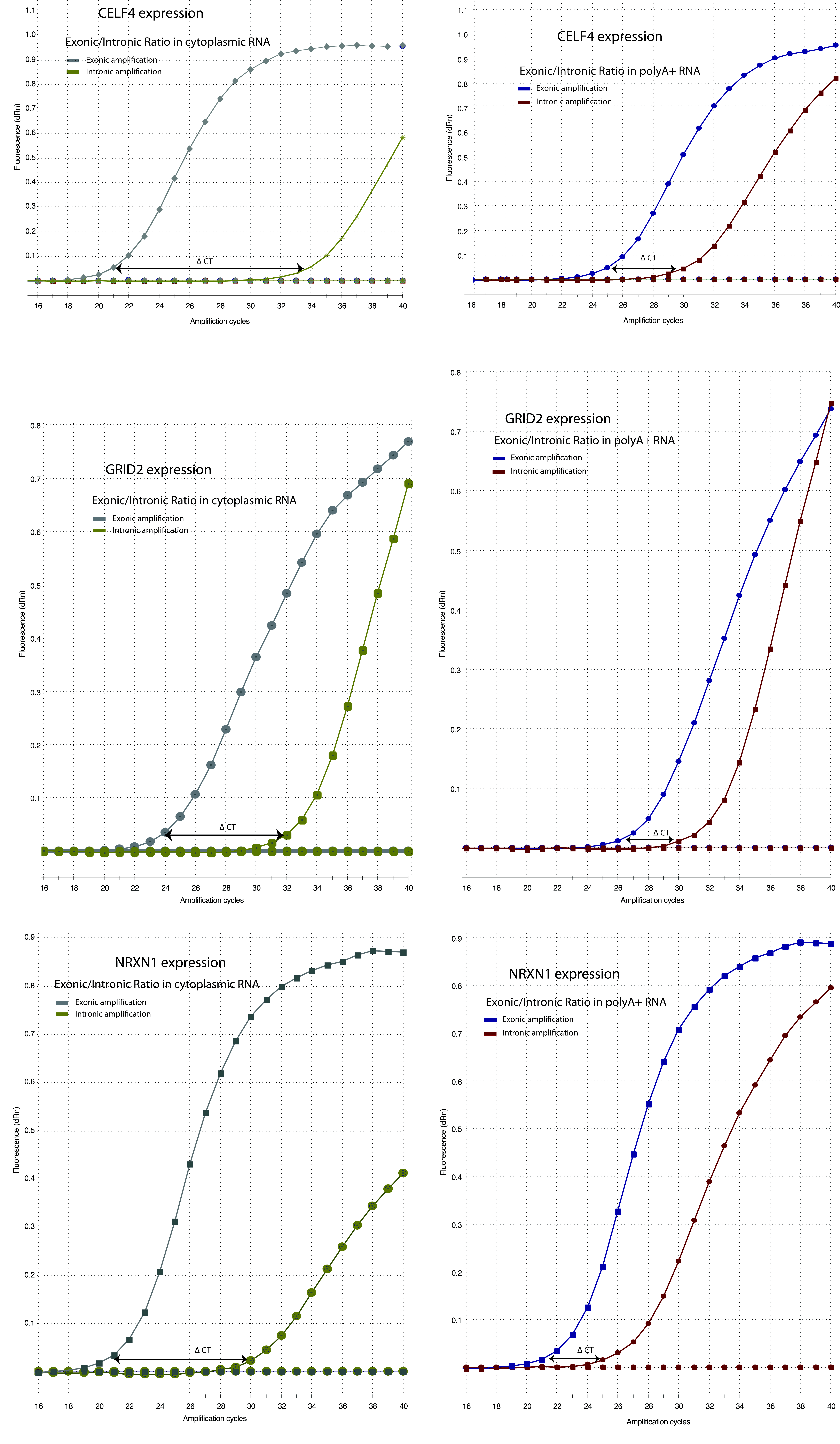
**

**Supplementary Figure S4.** CT values for exonic and intronic expression between the cytoplasmic and polyA+ selected RNA. QPCR charts, obtained from the MxPro Mx3000P software (Stratagene), showing the raw CT values differences (ΔCT ratio) between the exonic and intronic expression in *CELF4, GRID2* and *NRXN1* for cytoplasmic and polyA+ selected RNA populations.

**Supplementary Figure S5.** Cytoplasmic and nuclear RNA purified from brain tissue using the Cytoplasmic and nuclear RNA purification kit (Norgen) without modifications. A) Bioanalyzer analysis of cytoplasmic and nuclear RNA. Ribosomal RNA detected in the nuclear fraction indicating cross contamination from the cytoplasmic fraction. B) Agarose gel electrophoresis indicating the cross contamination between the nuclear and the cytoplasmic fraction. Genomic DNA and ribosomal RNA traces are detectable in both fractions. Samples 1 and 2 show results with modifications and samples 3 and 4 without modifications. C) qrtPCR showing higher retrieval of mature transcripts when modifications are added to the original Norgen protocol. The relative fold difference between exon expression and intron expression was measured in cytoplasmic fraction purified with and without modification. Primers were designed within an intron and the two surrounding exons for three genes (NRXN1, CELF4 and GRID2) according to the schematic representation in figure 2. ΔΔCT was first calculated to measure the exon/intron fold difference for each gene in the two purifications. The values obtained for the modified protocol were then normalized to the values obtained from Norgen protocol without modification. All samples were run in triplicate and expression values were normalized to the level of Beta-actin.

**Supplementary Tables**

**Supplementary Table S1. Cut-off values and number of expressed exons out of 353832 RefSeq exons**

|  | **Sample 1** | | | | **Sample 2** | | | |
| --- | --- | --- | --- | --- | --- | --- | --- | --- |
|  | **Cyt** | **PolyA** | **Total** | **Nuc** | **Cyt** | **PolyA** | **Total** | **Nuc** |
| **Cut-off v1**  **(dcpm)** | 0.0785 | 0.0785 | 0.0785 | 0.0785 | 0.0785 | 0.0785 | 0.0785 | 0.0785 |
| **Expressed**  **Exons v1** | 220659 | 203583 | 147622 | 137079 | 241704 | 203924 | 187836 | 168962 |
| **Cut-off v2**  **(dcpm)** | 0.0836 | 0.0975 | 0.0643 | 0.0589 | 0.0750 | 0.0863 | 0.0778 | 0.0843 |
| **Expressed**  **Exons v2** | 218550 | 190198 | 160094 | 158283 | 243132 | 198587 | 188233 | 165086 |

- v1 Cut-off for expression levels calculated from all samples togehter
- v2 Cut-off for expression levels calculated for each of the samples individually

**Supplementary Table S2.** Primer sequences for quantification of intronic and exonic expression in *NRXN1*, *CELF4* and *GRID2*.

| Primer name | Primer sequence |
| --- | --- |
| NRXN1INTR33 | CTTCAGTGGACCACTCTGCAAT |
| NRXN1INTF22 | GGCTGCAACAACTCAGTTCA |
| NRXN1INTR22 | GCTGAATTTGAATGTGGATT |
| NRXN1EXFF | CCACTTATACGTGATTTGTCCA |
| CELF4INTR1FOR | AACTGCTCTCTGGGACTCCA |
| CELF4INTR1REV | CAGCACATTAGGTGCAGAGC |
| CELF4EX1REV2 | GCAACCTGGATGAGAAGGAC |
| CELF4EX2FOR2 | CTGACTCACGCTCGCAGTAG |
| GRID2INF | AGAGTAGAACTTGAACTGAAGAG |
| GRID2INR | GCTTTCATGTTCCACCCAGA |
| GRID2U2R | TGCCATCAACAAACGTCACT |
| GRID2 5pexon1for | TCCCCTTTCTCTTGGTTTTG |
